# *Porphyromonas gulae* and PPAD antibodies are not related to citrullination in rheumatoid arthritis

**DOI:** 10.1007/s00784-023-04964-w

**Published:** 2023-05-03

**Authors:** Camilo Castellar-Mendoza, Diana Marcela Castillo, Lorena Chila-Moreno, Wilson Bautista-Molano, Consuelo Romero-Sánchez

**Affiliations:** 1 Master’s Program in Basic Biomedical Sciences, Universidad El Bosque, Bogotá, Colombia; 2 Oral Basic Research Unit (UIBO), Research Vice-Rectory, School of Dentistry, Universidad El Bosque, Av. Carrera 9 #131A-02, Bogotá, Colombia; 3 School of Dentistry, INMUBO Cellular and Molecular Immunology Group, Universidad El Bosque, Av. Carrera 9 #131A-02, Bogotá, Colombia; 4 School of Medicine, Clinical Immunology Group, Universidad Militar Nueva Granada/Hospital Militar Central, Transversal 3ª #49-00, Bogotá, Colombia; 5 Department of Rheumatology and Immunology/Clinical Immunology Group, Hospital Militar Central, Transversal 3ª #49-00, Bogotá, Colombia

**Keywords:** Rheumatology, Immunology, Bacteria, Periodontium, *Porphyromonas gulae*, Rheumatoid arthritis, Periodontitis, Antibodies

## Abstract

**Introduction:**

*Porphyromonas gulae* have the enzyme PPAD, as *P. gingivalis*, which is responsible for citrullination related to the pathophysiology of rheumatoid arthritis and periodontitis; this implies the presence of two species of PPAD-producing bacteria in the mouth as well as the presence of citrullinated proteins. There are no previous reports or studies investigating an association between *P. gulae* PPAD in rheumatoid arthritis (RA).

**Objective:**

To assess the presence of *P. gulae* and anti-citrullinated peptide antibodies of *P. gulae* PAD in patients with RA and their possible relationship with clinical activity markers.

**Subjects and methods:**

A total of 95 patients with RA and 95 controls were included. Erythrocyte sedimentation rate (ESR), C-reactive protein, anti-citrullinated protein antibodies (ACPAs) and rheumatoid factor (RF) were measured. Activity index-28 (DAS28) and SCDAI. The periodontal diagnosis was established. Presence of *P. gulae* and *P. gingivalis*. An ELISA was used to determine antibodies against citrullinated peptides of *P. gulae* PAD.

**Results:**

A *P. gulae* frequency of 15.8% was observed in the RA group and 9.5% in the control group. Higher levels of ACPA were found in the *P. gulae*-positive patients of the RA group, finding no significant difference, but if in patients positive for *P. gingivalis* with statistical significance (*p* = 0.0001). The frequency of anti-VDK-cit and anti-LPQ-cit9 antibodies to PPAD of *P. gulae* was higher in the RA group than in the control group without significant difference. No relationship was found with the clinical variables despite the presence of *P. gulae* and anti-citrullinated peptide antibodies of *P. gulae* PPAD in patients with RA

**Conclusions:**

It was not possible to establish a connection with clinical variables in RA and *P. gulae*; as a result, the presence of *P. gingivalis* continues to contribute significantly to the increase in antibodies against citrullinated proteins/peptides from exogenous sources of citrullination in RA and periodontitis.

**Supplementary Information:**

The online version contains supplementary material available at 10.1007/s00784-023-04964-w.

## Introduction

It has been determined that rheumatoid arthritis (RA), an autoimmune disease, affects 0.5–1% of the adult population in industrialised countries [1, 2]. Londoño et al. (2018) reported a prevalence of 1.49% (confidence interval 95%: 1.12–1.98%) in Colombia [3]. The disease affects women more than men (3:1), and its prevalence increases with age, peaking between the ages of 50 and 59 years [4]. Since it is an autoimmune and inflammatory disease, it involves several organs and systems and is characterised by persistent synovitis, destruction of cartilage and bone, synovial hyperplasia, recruitment of inflammatory cells and intra-articular fibrin deposition that leads to the destruction of joint architecture, loss of functionality and significant morbidity and mortality, in addition to disability [5]. Its aetiology is still unknown; however, genetic predisposition and the presence of certain infectious agents are thought to play a role in its pathogenesis [1, 2]. The autoimmune component of RA is characterised by the production of autoantibodies, mainly rheumatoid factor (RF) and anti-citrullinated protein antibodies (ACPAs) [6, 7].

According to Health Metrics, a group that investigates the economic impact of different risk factors based on death and years of life lost due to death or disability, RA increased the years of productive life lost due to disability by 82% between 1990 and 2010, with a total of 27,910 years in 2010. When years lost due to disability and death are added, the increase is 66% during the same period, with a total of 31.76 years [8].

Peptidylarginine deiminase (PAD) enzymes are important molecules in the pathophysiology of RA. They are a family of cysteine ​​hydrolase enzymes made up of five isoforms [1–4, 6]. These isoenzymes have an amino acid sequence homology of 70–90%, a weight of ~74 kDa and two N- and C-terminal domains with five calcium-binding sites (three located in the N-terminal domain and two in the C-terminal), where the active site of the enzyme is found. PADs are activated during regulated physiological processes, such as apoptosis and cell differentiation, in which calcium concentrations increase. A change in PAD regulation is associated with the development of diseases, including RA, Alzheimer’s, lupus, multiple sclerosis, Parkinson’s and cancer [9, 10]. Furthermore, autoimmune diseases associated with PAD enzymes are mainly characterised by the generation of autoantibodies. Citrullination is a key process in the pathophysiology of RA because it leads to the formation of antibodies against citrullinated proteins, including human PAD [7].

Currently, RA is strongly associated with periodontitis, primarily because of the inflammatory component and structural destruction; however, their relationship begins at the molecular level, where anaerobic, Gram-negative bacteria such as those of the genus *Porphyromonas* are protagonists [11]. Recently, it was described as the unique and crucial role of periopathogens in the initiation and progression of RA [12]. Periodontitis is a chronic and infectious pathology that destroys the supporting tissues of the teeth, including the gingival connective tissue, periodontal ligament and alveolar bone, and in a recent review shows about, not only epidemiological, but biologic association between these diseases [13, 14]. *P. gingivalis*, a Gram-negative, anaerobic, asaccharolytic, non-sporulating, non-motile coccobacillus bacterium, is most closely associated with periodontitis. It is a member of the black-pigmented Bacteroides group, the *Porphyromonadaceae* family and the *Bacteroidetes* phylum. The previously mentioned PAD enzyme, known in this bacterium as *Porphyromonas* PAD (PPAD), is one of its virulence factors [11]. This enzyme is associated with the stimulation of antibody formation against bacterial citrullinated proteins/peptides. This bacterium thus constitutes another source of citrullination [9, 10]. Additionally, it has been proposed a bidirectional relationship, because it is known that systematic factors may shape the periodontal microbiome, where different systemic and mechanistic bases modulate those relationships, and this has an impact over the management, treatment and evolution of patients [15].

*P. gulae* is another Gram-negative bacterium belonging to the family *Porphyromonadaceae* and is non-motile and non-spore-forming. In cultures, it produces black-pigmented colonies with asaccharolytic metabolism and obligate anaerobic growth. The virulence factors in its structure are similar to those of *P. gingivalis*, such as the presence of lipopolysaccharide (LPS), proteases that are similar to the gingipains of *P. gingivalis* and PPAD [12, 13]. This microorganism was first described in 2001 and isolated from the gingival sulci of mammals as part of the oral microbiota [16]. In companion animals, the presence of *P. gulae* is related to the development of periodontitis, a disease that induces bone inflammation and destruction [17]. *P. gulae* isolations have been recently reported from the oral cavity of humans living with domestic animals [18].

According to a 2018 study [19], not only *P. gingivalis* but also *P. gulae* and *P. loveana* are producers of PPAD and are also homologous to each other (up to 93%), including residues of the amino acids from catalytic sites of the deamination reaction. They also protect PPAD functions, including protecting *Porphyromonas* proteins from degradation by gingipains via citrullination and participating in post-translational changes that allow gingipain activation [19]. Moreover, the citrullination of host proteins allows for the neutralisation of acidic environments via the production of ammonia as a by-product of the citrullination reaction, which favours their proliferation [19]. *P. gulae* has morphological and genetic characteristics similar to those of *P. gingivalis* and is the cause of periodontitis in domestic animals (dogs and cats) [14]. However, due to the practice of keeping pets as part of the family, the transmission of *P. gulae* from dogs to humans was detected in all cases where the bacteria were detected in the animal [20]. This implies the presence of two species of PPAD-producing bacteria in the mouth as well as the presence of citrullinated proteins (both bacterial and host), which are closely associated with the initiation and establishment of RA [21].

Recently, a study about the role of *P. gulae* proteinases in bacterial and host cell biology showed an ability to agglutinate mouse erythrocytes, co-aggregation with *Actinomyces viscosus*, inhibition of Ca9-22 cell proliferation in a multiplicity of infection and time-dependent manner and induction of decrease in cell contact and adhesion. All these results indicate suppression of the amount of human proteins, such as γ-globulin, fibrinogen and fibronectin, by *P. gulae* proteases, suggesting that a novel protease complex contributes to bacterial virulence [22].

This investigation evaluates the presence of *P. gulae* and *P. gingivalis* in subgingival plaque samples collected from patients with RA and systemically healthy individuals. We also evaluated the presence of anti-citrullinated peptide antibodies of *P. gulae* PAD protein in serum samples taken from patients with RA and healthy controls. Whether there is an association between the presence of *P. gulae*, *P. gingivalis* and clinical markers of RA was also evaluated.

## Materials and methods

A cross-sectional study was performed on individuals with RA between the years 2015 and 2018. Two groups of subjects were included in this study: 95 individuals with RA and 95 healthy individuals (control group) matched for gender and age. The inclusion criteria for the RA group were subjects aged 18–65 years with classification criteria for RA according to the American College of Rheumatology and EULAR of 2010 [23] and established RA with >2 years of disease progression. Individuals aged 18–65 years who were in similar work or environmental circumstances and had no family ties to patients with autoimmune diseases were included in the control group. The exclusion criteria for both groups were as follows: individuals with an ongoing infection, diagnosis of an autoimmune disease other than RA (in cases group), periodontal treatment in the last 6 months, antibiotic use in the last 3 months, breastfeeding or pregnant, cancer or diabetes. This study was approved by the Ethics Committees of Hospital Militar Central, Bogotá-Colombia 2016-04/2016-012 and Universidad El Bosque, Bogotá-Colombia. All participants signed informed consent forms prior to their participation.

### Biomarkers of inflammation and autoantibodies

Rheumatologists measured the level of RA disease activity using the disease activity score 28 (DAS28) [24] and simplified disease activity index (SDAI) [25]. Chemiluminescence technology (IMMULITE 1000, Siemens®, Berlin-Germany) was used, with values ​​in the normal range between 0 and 3 mg/L for the quantification of C-reactive protein (CRP). ACPAs IgG (immunoglobulin G)/IgA levels in the serum were measured quantitatively by an enzyme-linked immunosorbent assay (ELISA) (QUANTA Lite® CCP 3.1 IgG/IgA, INNOVA Diagnostics, Irvine, CA, USA), with ranges ≥20 IU/mL considered positive. The RF measurements were evaluated using the kinetic turbidimetry technique (Spinreact®, Sant Esteve d’en Bas (GI), Spain), with values ≥20 IU/mL considered positive, and the erythrocyte sedimentation rate (ESR) was determined using quantitative capillary photometry technology (Alifax Spa ®, Padova, Italy) (normal value < 20 mm/h). All tests were performed in accordance with the manufacturers’ instructions.

### Periodontal evaluation

All patients were diagnosed with periodontitis according to the criteria of the Centers for Disease Control and Prevention [25]. The clinical indexes were taken by two periodontists who participated in an inter-examination calibration. In addition, repeated evaluations were conducted before the study on 5 randomly selected subjects in order to determine intra-examiner reproducibility. The clinical indices used were pocket depth (PD), intra-examiner intraclass correlation coefficient (IE-ICC 0.92 to 0.98), clinical attachment level (CAL) (IE-ICC 0.90 to 0.98), bleeding on probing (BoP) (IE kappa index 0.85 to 0.95), the percentage of visible plaque on the dental surfaces in full mouth (PI) (IE-kappa index 0.85 a 0.92) and gingival index (GI) (IE-kappa index 0.88 to 0.94) [26] A full mouth examination was performed, which included selected sites on each permanent tooth, excluding the third molars.

### Detection of *P. gingivalis*

*P. gingivalis* was detected using the quantitative polymerase chain reaction (qPCR) technique. TaqMan primers and probe were used as described by Boutaga et al. [27]. PCR amplification was performed in a total reaction volume of 25 μL. The reaction mixture contained 3 mM MgCl2, 1X GoTaq Polymerase® buffer, 0.1 mM dNTP, 0.9 μM *P. gingivalis*-specific probe, 1 μM primers and 0.125 U GoTaq Polymerase® (GoTaq Polymerase® [Promega], Madison, WI, USA). The samples underwent an initial amplification step at 95°C for 10 min, followed by 45 cycles at 95°C for 15 s and 60°C for 1 min in a Bio-Rad™ CFX96 thermocycler. Quantification was based on a calibration curve with known amounts of DNA from *P. gingivalis* ATCC 33277 expressed in CFU. The data was converted to log10 prior to performing statistical analysis [11].

### Detection of *P. gulae*

Briefly, the presence of *P. gulae* bacteria in subgingival plaque samples was evaluated using qPCR. The sense primer reported by Senhorinho et al. in 2011 (5′-TTGGTTGCATGATCGGG-3′) [28] was used. The antisense primer (5′-TACGGGAGGCAGCAGTG-3′) and TaqMan probe (5′-FAM-AAGGCTACGATGGGTAGGG -3′ BHQ1) were specifically designed for this study. A more detailed description is mentioned in Appendix Supp information 1 and Figure S1.

## Prediction and selection of citrullinated peptides from *P. gulae* PAD

In summary, eight peptides of the *P. gulae* PAD enzyme was synthesised, including two native peptides (VDK and LPQ), four peptides with modified structure and citrulline residue in different positions (VDK-cit, LPQ-cit9, LPQ-cit15 and LPQ-cit9/15) and two peptides modified in the order of the amino acid sequence (VDK-random and LPQ-random). A more detailed description is mentioned in Appendix Supp information 2, Fig. S2 and Fig. S3.

### Detection of anti-PAD antibodies of *P. gulae*

Serum *P. gulae* PAD (IgG) antibodies were detected using an in-house ELISA system. A more detailed description is mentioned in Appendix Figure S4.

### Statistical analysis

Sample size was calculated based on a previous pilot study using the TM® program, with a statistical power of 80% and an alpha error of 5% for 10 discordant pairs and an OR of 4 based on the preliminary calculation, resulting in a total of 90 individuals per group. Chi-square and Fisher’s exact tests were used for the analysis of categorical variables, such as demographic, rheumatological and periodontal data. The Kruskal–Wallis and Mann–Whitney *U*-tests for non-parametric data were used for the association of quantitative variables in two groups. All analyses were conducted using SPSS V24 and STATA for Windows software. The significance level was set at *p* ≤ 0.05. Furthermore, a discriminant analysis of multiple correspondences that allows the grouping of variables with high correlation coefficients (CC) was carried out. The results are displayed on a Cartesian plane, with the variables represented as vectors whose angles become more closed as the level of correlation between them increases. The length of each vector represents the correlation coefficient of the variable within the group, which ranges from −1.0 to +1.0. A high contribution was considered when the CC values were >0.7, intermediate when they were between 0.5 and 0.7 and low when they were <0.5 [29].

## Results

### Demographic data of the RA and control groups

The RA and control groups had a mean age of 47.32 ± 10.4 years and 47.85 ± 10.4 years, respectively. There was a higher proportion of women in both groups (78.90%). In addition, 6.3% of the participants in the RA group and 7.3% in the control group reported current smoking habits; 32.6% of the individuals in the RA group and 26.3% in the control group had a history of smoking; 12.6% of the individuals in the RA group and 17.9% in the control group were passive smokers; 40% of the individuals in the RA group and 27.4% in the control group were overweight; and 9.5% of the individuals in the RA group and 5.3% in the control group were obese.

The 55.7% were being treated with only conventional disease-modifying therapy (DMARD); the conventional DMARDs included were methotrexate (10–15 mg/week), leflunomide (20 mg/day), hydroxychloroquine (200 mg/day) and sulfasalazine (1.0–1.5 g/day); methotrexate was used in 86.8% of patients, and 44.2% received tumour necrosis factor as inhibitors of biological treatment simultaneously with DMARD, and the use of corticosteroids was reported at 60.0%.

### Serological and joint variables in the RA and control groups

Higher levels of ESR (23.83% ± 25.7), CRP (15.42% ± 29.22) and RF (98.56% ± 141.51) and a higher frequency of ACPA were observed in the RA group (438.69% ± 769.58) than in the control group. Similarly, the RA group had a higher number of individuals with painful joints than the control group (73.70% vs. 24.2%), as well as a higher number of swollen joints (63.2% vs. 8.4%) (Table 1).

**Table 1:** Serological and joint variables in the RA group and healthy controls

Variable		Control	RA
		Average ± SD	Average ± SD
ESR		14.91 ± 15.57	23.83 ± 25.7*
CRP		3.67 ± 4.79	15.24 ± 29.22*
RF		1221 ± 15.09	98.56 ± 141.51*
ACPAs (IgG/IgA)		11.22 ± 22.96	438.69 ± 769.58*
Frequency in percentage (%)			
DAS28	≤ 2.6	28.4%	69.5%*
	2.6–3.2	12.6%	13.7%
	>3.2	58.9%	16.8%*
SDAI	≤ 3.3	36.8%	6.3%
	3.3–11	45.3%	26.3%
	>11	17.9%	67.4%*

Chi-square test and Fisher’s exact test. *p* < 0.05*. *ESR* erythrocyte sedimentation rate, *CRP* C-reactive protein, *RF* rheumatoid factor, *ACPAs* anti-citrullinated protein antibodies, *DAS28* disease activity score 28, *SDAI* simplified disease activity index

### Periodontal variables in the RA and control groups

The frequency of periodontitis in the RA group was similar to that of the control group (70.50% for both groups), as was the frequency of *P. gingivalis* (52.60% vs. 51.60%, respectively), with no statistically significant difference. The severity of the disease also had a homogeneous behaviour in both groups. Conversely, IgG1 and IgG2 antibodies against *P. gingivalis* were more frequent in the control group compared to the RA group (Tables 2 and 3).

**Table 2:** Periodontal variables in the RA group and healthy controls

Variable		Control	RA
		Frequency (%)	
Periodontal disease	Absence	28 (29.5)	28 (29.5)
	Presence	67 (70.5)	67 (70.5)
Severity	None	28 (29.5)	28 (29.5)
	Mild	10 (10.5)	12 (12.6)
	Moderate	44 (46.3)	41 (43.2)
	Severe	13 (13.7)	14 (14.7)
*P. gingivalis*	Absence	46 (48.4)	45 (47.4)
	Presence	49 (51.6)	50 (52.6)
*P. gulae*	Absence	81 (90.5)	76 (80.0)
	Presence	9 (9.5)	15 (15.8)
A/b-IgG1-anti P.g ≥ 1/100	<1/100	37 (38.9)	49 (51.6)
	≥1/100	58 (61.1)	46 (48.4)
A/b-IgG2-anti P.g ≥ 1/100	<1/100	33 (34.7)	54 (56.8)
	≥1/100	62 (65.3)	41 (43.2)*

Chi-square test and Fisher’s exact test. *p* < 0.05*. *A/b* antibodies, *P.g P. gingivalis*

**Table 3:** Periodontal index in the RA group and healthy controls

Variable		Control	RA
		Median (IQR)	
% Plaque		0.56 (0.33–0.69)	0.57 (0.36–0.81)
%Gingival bleeding		0.41 (0.27–0.62)	0.31 (0.18–0.46)
Pocket depth	4–5 mm	2.00 (0.00–6.00)	3.00 (1.00–9.00)
	>5 mm	0.01 (0.00–0.02)	0.00 (0.00–0.02)
Attachment level	>2 mm	62 (35–78)	49 (28–79)
	>3 mm	30 (15–48)	21 (7–52)
	>4 mm	12 (13–29)	7 (1–22)
	>5 mm	3 (0–8)	2 (0–8)

### Comparison of the presence of *P. gulae* and *P. gingivalis* between the RA and control groups

**Fig. 1:**
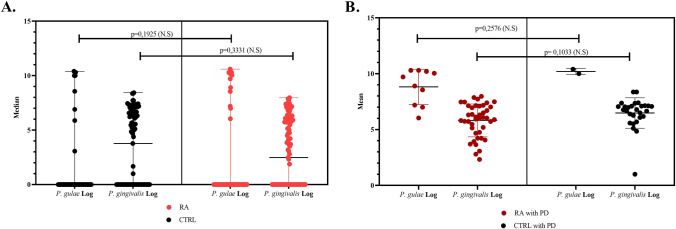
**A** Presence of *P. gulae* among patients with RA and healthy controls (CTR). **B** Presence of *P. gulae* in patients with RA and healthy controls with a diagnosis of periodontitis (PD). The values are expressed in log10. The data was analysed using the Mann–Whitney *U*-test. N.S. indicates no statistical significance

A higher frequency of *P. gulae* was observed in the RA group than in the control group (15.8% vs. 9.5%, respectively), without statistically significant differences (*p* = 0.1925). Similar results were obtained when the same comparison was made between the groups with *P. gingivalis* (*p* = 0.331). Comparisons were made by classifying the patients according to periodontal diagnosis. The presence of *P. gulae* was observed more frequently in the RA group with a diagnosis of periodontitis compared to the control group (6.7% vs. 2.5%, respectively), without statistical significance (*p* = 0.2576). Similar results were obtained when performing the same analysis with *P. gingivalis*, and no statistically significant difference was observed (*p* = 0.1033). Additionally, the mean and median of the quantification of *P. gingivalis* expressed in log10 were compared, and no statistically significant differences were observed (Fig. 1a and b).

### Presence of anti-citrullinated peptide antibodies of *P. gulae* PAD

**Fig. 2:**
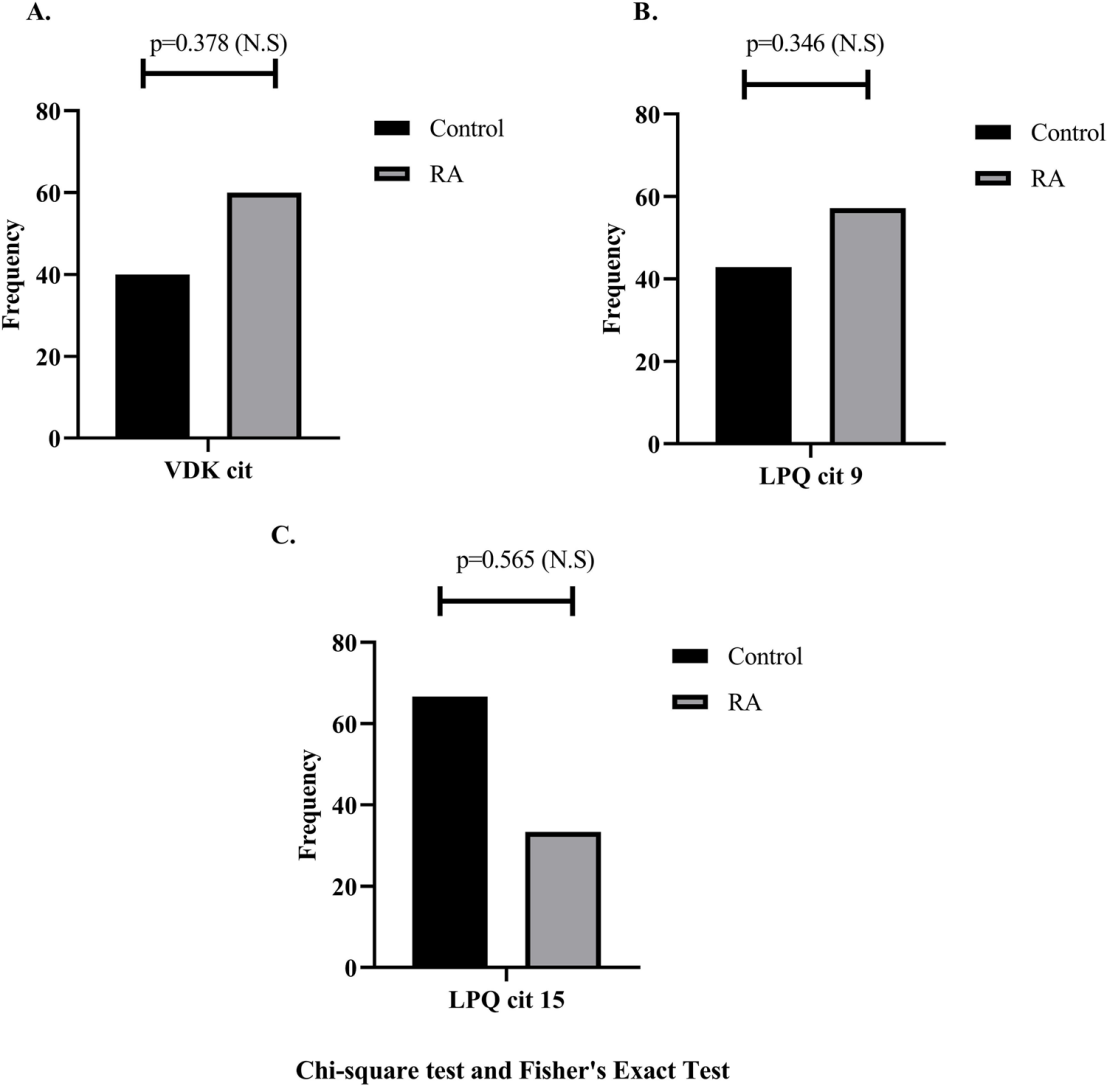
Frequency of anti-citrullinated peptide antibodies of *P. gulae* PAD. **A** VDK citrullinated, **B** LPQ citrullinated position 9 and **C** LPQ citrullinated position 15 in patients with RA and healthy controls (CTR). Chi-squared test (N.S not significant meaning)

Out of the 190 individuals evaluated, 18 (9.47%) had antibodies against one citrullinated peptides of *P. gulae* PAD. The frequency of anti-VDK-cit and anti-LPQ-cit9 antibodies was higher in the RA group (60% and 57%, respectively) than in the control group (40% and 42%, respectively), with no statistically significant difference (*p* = 0.378 and *p* = 0.346, respectively). Conversely, the frequency of anti-LPQ-cit15 antibodies was higher in the control group (66%) than in the RA group (34%), with no statistically significant difference (*p* = 0.565) (Fig. 2).

**Fig. 3:**
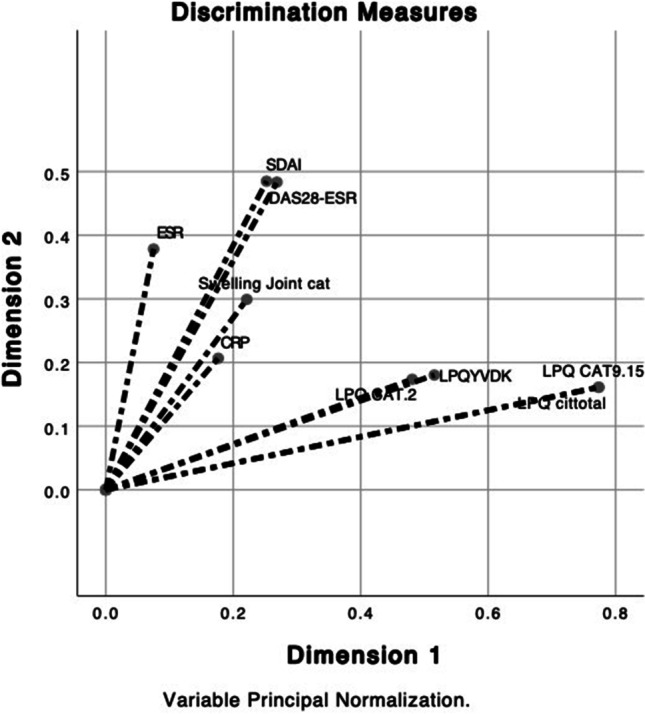
Discriminant analysis of multiple correspondences between clinical and laboratory variables of RA and the presence of anti-citrullinated peptide antibodies of *P. gulae* PAD. ESR erythrocyte sedimentation rate, SDAI simplified disease activity index, DAS28 disease activity score 28. No positive correlation was found between the clinical variables of RA and anti-citrullinated peptide antibodies of *P. gulae* PAD

Furthermore, the presence of antibodies against native peptides of *P. gulae* PPAD, that is, peptides that have not been modified, was evaluated in order to determine the naive immunogenic capacity of each of them, but positive results were not observed. Multiple correspondence discriminant analysis revealed two dimensions: one comprised of the clinical activity variables of RA (SDAI CC, 0.258; DAS28VSG CC, 0.261; ESR CC, 0.087; and swollen joints CC, 0.218) and a second dimension in which the presence of antibodies against citrullinated peptides of *P. gulae* PAD (anti-LPQ-cit9/15 CC, 0.749; anti-LPQ-cit total CC, 0.749; anti-LPQ/VDK CC, 0.520; and anti-LPQ-cit 9 CC, 0.476) was related to each other. However, no positive correlation was found between the clinical variables of RA and anti-citrullinated peptide antibodies of *P. gulae* PAD (Fig. 3); we did not obtain any cross-reactivity against citrullinated peptides of *P. gingivalis* PAD.

### Association between the presence of *P. gulae* and *P. gingivalis* and clinical markers of RA

**Fig. 4:**
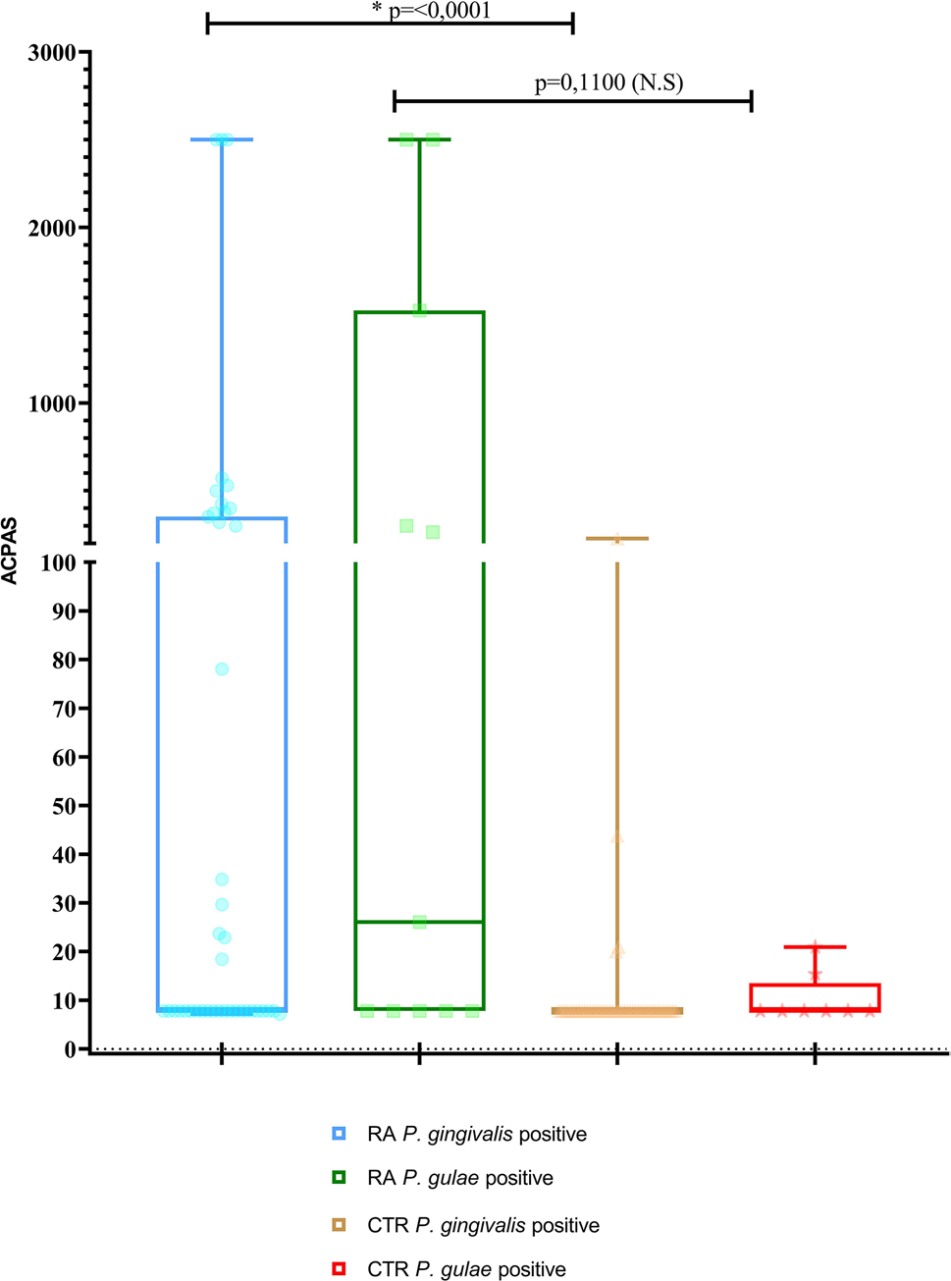
Association between the presence of *P. gulae* and anti-citrullinated protein antibodies (ACPAs) in patients with RA and healthy controls (CTR). Mann–Whitney *U*-test. Asterisk indicates statistical

Higher levels of ACPA were found among the *P. gulae*-positive patients of the RA group with respect to the positive *P. gulae* individuals of the control group, although no statistically significant differences were found (*p* = 0.110). The same was observed between positive *P. gingivalis* patients in the RA and control groups, with statistical significance (*p* = 0.0001) (Fig. 4).

Otherwise, we analyse the concentrations of *P. gulae* in the RA group versus the periodontal diagnosis, and we found statistically significant differences, finding a higher bacterial quantity in those without PD (*p*=0.014).

**Fig. 5:**
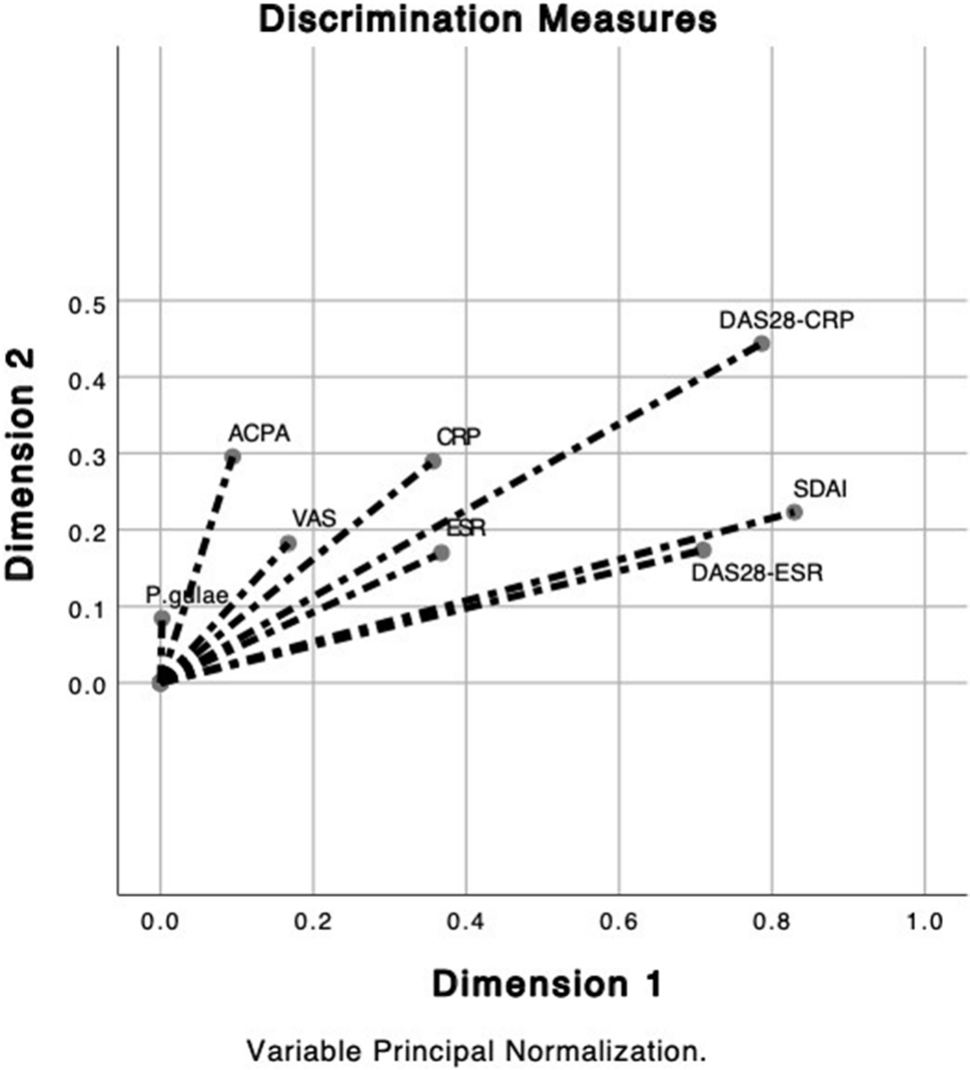
Discriminant analysis of multiple correspondences between the clinical and laboratory variables of RA and the presence of *P. gulae* in patients with RA. RF rheumatoid factor, CRP C-reactive protein, ACPA anti-citrullinated protein antibody, VAS visual analogue scale, ESR erythrocyte sedimentation rate, SDAI simplified disease activity index, DAS28 disease activity score 28. No positive correlation was found between the clinical variables of RA and *P. gulae*

Finally, similar to the previous result, multiple correspondence discriminant analysis revealed a dimension characterised by the presence of *P. gulae* with no significant correlation with the presence of ACPA, CRP, ESR, DAS28PCR, SDAI and DAS28VSG (CC: 0.186) (Fig. 5).

The analyses of RA/*P. gulae* positive and anti-PPAD peptides of *P. gulae* versus ACPAs do not show significant differences with the level of the ACPAs to indicate the influence of these antibodies on the positivity of the ACPAs.

## Discussion

Isolates of *P. gulae* have been obtained from the gingival sulci of different mammalian species, including bears, opossums, dogs, cats, coyotes, kangaroos, monkeys, sheep and wolves [30–32]. Furthermore, it was isolated in greater proportions from the gingival sulci of dogs with periodontitis than from healthy animals [16, 30]. There are recent reports on the presence of *P. gulae* in healthy human gingival tissues and those with periodontitis [17, 31], including a study demonstrating the induction of inflammatory response and decreased cell motility in human cell lines due to *P. gulae* infection [33]. The results of this study indicate colonisation by *P. gulae* in patients with RA and in healthy controls, although to a lesser extent than the quintessential periodontal species, *P. gingivalis*. However, *P. gulae* should not be ruled out as a potential contributor to infection and inflammatory processes leading to periodontitis in these population groups. We found no previous reports of *P. gulae* colonisation in periodontal pockets of patients with RA to compare our results. We do not have complete information, at the time when these patients were included. However, retrospectively, it was possible to obtain information from 60% of the total group, obtaining a frequency of 40% of living with pets.

It is remarkable that 9.47% (9/95) of the control subjects presented with *P. gulae* and concomitantly 44.4% (4/9) with *P. gingivalis*, without finding a diagnosis of periodontitis in any of these subjects. This was in contrast to the group of patients with RA who presented with *P. gulae* in 15.8% (15/95) and concomitantly *P. gingivalis* in 53.3% (8/15), and seven were clinically classified with a diagnosis of periodontal disease in 87.5%, potentially influencing the susceptibility of the host; besides, the higher bacterial quantity in those without PD may suggest that the contribution of this bacterium is not decisive like that of *P. gingivalis* or that the *P. gulae* needs the contribution of other risk factors to influence the periodontal diagnosis. The above needs to be evaluated in a larger number of subjects.

Based on its genetic and phenotypic homology with *P. gingivalis*, including the expression of virulence factors such as fimbriae, it was demonstrated that *P. gulae* has the capacity to invade human gingival epithelial cells and the mechanism used by this bacterium for invasion may be similar to that of *P. gingivalis* [34]. However, host specificity and subsequent pathogenicity may explain the difference in the distribution of the two periodontopathic species, which is supported by the distinct separation of the *fimA* and *mfa1* genotypes between these microorganisms [35].

Inflammation is the common clinical mechanism between RA and periodontitis, but there are also strong epidemiological, serological and clinical associations [36]. Indeed, the chronic stage of periodontitis is directly correlated with unusually high levels of ACPA; thus, it may contribute to the inflammatory reaction of RA and vice versa [36, 37]. It has been reported that patients with RA have twice the prevalence of periodontitis, indicating a positive feedback loop between the two diseases [11, 35, 37]. These results were not found in our study group, although the patients had more than 5 years of progression. Similar results were described by Mikuls et al. [38].

Of the five human PAD isoforms, increased levels of PAD2 and PAD4 are found in localised pathologic sites of RA and periodontitis (joints and periodontal pockets), leading to increased protein citrullination [39], and, as mentioned before, *P. gingivalis* (a key periodontitis pathogen) secretes a PAD called PPAD [40–42] as a virulence factor. Therefore, during tissue destruction in infected gums, PPAD generates new epitopes for the recognition of citrullinated proteins/peptides in susceptible individuals, resulting in increased inflammation, with citrullination becoming one of the common biological and molecular mechanisms in RA and periodontitis. Overall, this finding led to PPAD being considered an aetiological factor for RA [43, 44] and a promising therapeutic target against periodontitis and RA. Also, considering that PPAD undergoes autocitrullination processes, it additionally becomes a source of anti-citrullinated peptide antibodies [45]. The study conducted by Gabarrini et al. revealed the presence of PAD in other *Porphyromonas* species as well as the identity of 93% of the amino acid sequence of *P. gingivalis* PPAD with its homologue of *P. gulae*, as well as the conservation of the classification pattern of the PPAD enzyme towards the extracellular environment between species, suggesting a biological and/or clinical relevance [19]. Our results demonstrate the generation of antibodies against citrullinated peptides of *P. gulae* PAD in patients with RA and controls. Therefore, using the previously proposed reciprocity model between RA and periodontitis, we were unable to demonstrate that the presence of *P. gulae* represents another source of exogenous citrullination. It should be noted, however, that none of the subjects, both in the RA and control groups, who had antibodies against modified peptides had a response against native peptides. This demonstrates the low immunogenic capacity of the original protein and suggests the importance of citrullination in the generation and increase of ACPAs in its early stages.

The findings demonstrate the presence of autoantibodies associated with RA in both patients with RA and controls. Contrary to what the name suggests, RF are found not only in RA but in a wide range of pathologies including other autoimmune and non-autoimmune diseases. They have been found in up to 4% of young, healthy individuals and the elderly as well [46].

Rheumatoid factors are not routinely detectable in the circulation without an immunogenic stimulus. They are considered to be part of the normal response to a variety of antigenic stimuli, for example, to bacterial toxins like lipopolysaccharides or viruses such as Epstein–Barr virus (EBV) [47]

They form immune complexes that are subsequently phagocytosed by the inflammatory cells. These RFs are low-affinity, transient and polyclonal antibodies produced from the germinal centre. Their role could be considered protective in this context in the absence of disease [48, 49].

The structure of PPAD is only remotely related to human PADs, and although they are both endodeiminases, PPAD is a C-terminal exodeiminase [50]. It is unknown whether *P. gulae* PAD in its citrullination mechanism also requires the arginine residue susceptible to post-translational modification to be at the C-terminus of the peptide sequence as proposed in *P. gingivalis* [40, 41]. However, our results, in which the highest frequency of recognition with antibodies was towards the PAD peptides from *P. gulae* with a citrulline residue in the centre of the sequence, indicate that the position of the modification is probably important in the generation of the epitope and the subsequent immune response with the generation of antibodies. It is also worth noting that none of the three citrullinated peptides were recognised simultaneously (Fig. 2b). There is evidence that *P. gingivalis* PPAD has citrullinating activity in the gingival tissue, resulting in antibody formation [45]. However, no reports of the same with *P. gulae* PAD have been found.

In this study, we do not consider other sources of citrullinated proteins, such as NETosis, a chromatin meshwork edged with antimicrobial peptides usually present in neutrophil granules. This phenomenon is enhanced in the synovium patients with RA and microbial and inflammation diseases, like periodontitis; this situation could create another perpetual vicious circle of production of citrullinated proteins and, consequently, ACPA [12].

To the best of our knowledge, there are no investigations to date that evaluate the presence of *P. gulae* in patients with RA and its possible role in the pathophysiology of this autoimmune-inflammatory disease. The association between *P. gingivalis* and the level of ACPA, which are markers of risk, diagnosis and progression of RA [45], was confirmed once more, as levels of these antibodies were higher in patients with RA and in the presence of *P. gingivalis* than in controls. To date, the findings in this group of patients with RA and in the presence of *P. gulae* do not lead us to consider *P. gulae* as a direct source of exogenous citrullination and a contributor to the reciprocal and biological mechanisms common in RA and periodontitis.

## Conclusion

No relationship was found with the clinical variables despite the presence of *P. gulae* and anti-citrullinated peptide antibodies of *P. gulae* PPAD in patients with RA, which could be an indication of a minor contribution of this microorganism to the biological and molecular mechanism of citrullination, common in RA and periodontitis. Therefore, the presence of *P. gingivalis* continues to contribute significantly to the increase in antibodies against citrullinated proteins/peptides from exogenous sources of citrullination in patients diagnosed with RA and periodontitis.

This is the first study to evaluate another pathogen as a possible exogenous source of citrullination in the synergistic model between RA and periodontitis in a population with RA and systemically healthy controls, generating significant knowledge in this same reciprocal model between chronic diseases. The results were obtained in a single clinical moment, and the sample size for both study groups were limited, implying the need for further studies and follow-up, as well as increased patient and control participation.

## Supplementary information

ESM 1

## Data Availability

The data that support the findings of this study are available upon request from the corresponding author.
