# Supplementary material for: Porphyromonas gulae and PPAD antibodies are not related to citrullination in rheumatoid arthritis
Source: Clin Oral Investig. 2023 May 3;27(7):3509–19. doi: 10.1007/s00784-023-04964-w (PMC10329627; doi:10.1007/s00784-023-04964-w)
Supplement: Supplementary file 1 — ESM 1 [file 784_2023_4964_MOESM1_ESM.docx]

**Supplementary material: The presence of *Porphyromonas gulae* and anti-PAD/*P. gulae* antibodies in patients with rheumatoid arthritis is not related to exogenous citrullination or disease activity**

**Castellar-Mendoza C, Castillo DM, Chila-Moreno L, Bautista-Molano W, Romero-Sánchez C**

**Supp information 1: Quantitative polymerase chain reaction (qPCR) for the detection of *Porphyromonas gulae* in samples from patients with rheumatoid arthritis (RA) and healthy controls**

Specificity was verified because there were previously reported primers, and we confirmed that these primers could amplify both species due to high homology between *P. gingivalis* and *P. gulae*. Therefore, we decided to use the sense primer reported by Senhorinho et al. in 2011 (5′-TTGGTTGCATGATCGGG-3′) (1). The antisense primer and TaqMan-type probe were designed using the sequence reported in GenBank that corresponds to the gene that codes for 16S rRNA and using the Primer3Plus program (http://primer3plus.com/). Once the sequences were obtained, the specificity was confirmed using Primer-BLAST (http://www.ncbi.nlm.nih.gov/tools/primer-blast/), in addition to *in silico* PCR and a manual review, to avoid cross-reactions between the two species mentioned above.

For qPCR standardisation, DNA extraction was performed from the *P. gulae* ATCC 51700 strain and the genus *Porphyromonas* strains of oral interest available in the Oral Microbiology Laboratory strain collection of the UIBO Institute (Research Unit Basic Oral), Universidad El Bosque: *P. gingivalis* ATCC (American Type Culture Collection) 33277, *P. gingivalis* W83, *P. levii* ATCC 29147 and *P. endodontalis* ATCC 35406. The DNA of *P. gulae* ATCC 51700 was extracted using two different methods: an in-house method involving thermal shock and another involving the Qiagen™ kit (40724 Hilden, Germany). The extraction method used for the other strains was the in-house thermal shock method.

A conventional PCR amplification was initially performed to determine the concentrations of reagents for the PCR mixture and of the primers using *P. gulae* ATCC 51700 (5 µL) DNA extracted. The final reagent concentrations were 2.5 µM MgCl2 (Magnesium chloride), 0.25 µM dNTPs, 25 UI Taq polymerase and 4 µM primers. The amplification protocol for 34 cycles was 95°C/3:00 min, 95°C/00:30 s, 53°C/00:30 s, 72°C/01:00 min and 72°C/05:00 min. A BIO-RAD T100™ Thermal Cycler was used for this amplification process. The PCR products were analysed using 2% agarose gel electrophoresis. The agarose gel was then stained with ethidium bromide and visualised on a BIO-RAD Gel Doc XR+™ image analyser. A single band at ~173 bp can be seen in all the lanes in the agarose gel corresponding to *P. gulae* ATCC 51700 DNA extracted using two different techniques. No band can be seen in the negative control lane (Figure S1).


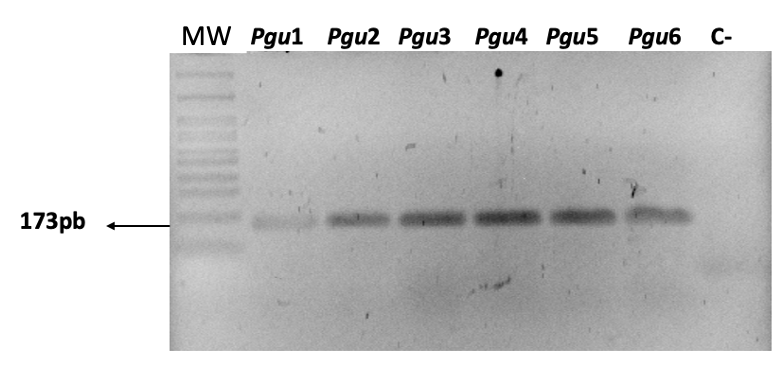


**Figure S1.** 2% agarose gel. A single band is observed at a height of 173 bp in the lanes with DNA from P. gulae ATCC 51700. MP: molecular weight marker; Pgu1, Pgu2, Pgu3, Pgu4: P. gulae ATCC 51700 DNA (heat shock extracted); and Pgu5, Pgu6: P. gulae ATCC 51700 DNA extracted using a Qiagen™ commercial DNA extraction kit. C-: negative control.

Similarly, PCR was performed in accordance with the previous amplification protocol to evaluate the specificity of the primers of *P. gulae* ATCC 51700, as a template, in addition to the DNA of *P. gulae* ATCC 51700, *P. gingivalis* ATCC 33277, *P gingivalis* W83, *P. levii* ATCC 29147 and *P. endodontalis* ATCC 35406. A single band is observed in the agarose gel image at the expected height (~173 bp) in the lanes corresponding to *P. gulae* ATCC 51700 negative control, but no bands are seen in the lanes corresponding to *P. gingivalis* ATCC 33277 and *P. gingivalis* W83. While a faint band is observed in the lanes of *P. levii* ATCC 29147 and *P. endodontalis* ATCC 35406, which are both at the same height, it does not correspond to the expected weight when the primers for *P. gulae* ATCC 51700 are used. Quanta™ buffer (Quantabio Suite 407J, Beverly, MA 01915) was used for qPCR. Primers and FAM-labelled TaqMan probe from *P. gulae* ATCC 51700 were used at a final concentration of 2 µM. The amplification protocol for 40 cycles was 95°C/03:00 min, 95°C/00:30 s, 53°C/00:30 s (detection) and 72°C/01:00 min. qPCR was performed on the CFX96™ Real-Time System of BIO-RAD. To avoid errors and possible pipetting contamination, five qPCR experiments were conducted on different days to evaluate the specificity of the primers and probes. In each one, apart from the negative control, DNA from the strains *P. gingivalis* ATCC 33277, *P. gingivalis* W83, *P. levii* ATCC 29147 and *P. endodontalis* ATCC 35406 (2 uL) was used. In the graphs of the amplification curves by relative fluorescence units, it was observed that in the wells corresponding to each strain of *Porphyromonas*, except for *P. gulae* ATCC 51700, there was no amplification curve, thus verifying the *in vitro* specificity of the sequences used in the design of the primers and the *P. gulae* ATCC 51700 probe. The same result was obtained for the strains *P. gingivalis* W83, *P. levii* ATCC 29147 and *P. endodontalis* ATCC 35406 (Figure S2). A calibration curve containing known amounts of DNA from *P. gulae* ATCC 51700 was used for quantification. The points of the curve were obtained from an inoculum of the bacteria obtained at an optical density of 535 nm, and subsequently, three replicates of the inoculum were made with serial dilutions with base 10 and DNA extraction by thermal shock (Figure S3).

**Supp information 2: In-house enzyme-linked immunosorbent assay (ELISA) for the detection of anti-citrullinated peptide antibodies of *P. gulae* peptidyl arginine deiminase (PAD) in samples from patients with RA and healthy controls**

The sequences of the PAD enzyme from *P. gulae* were analysed by homology using the National Center for Biotechnology Information databases (http://www.ncbi.nlm.nih.gov/genome/) and BLAST tools. According to the results, the B and T epitopes for this enzyme were predicted based on the location of the arginines within the molecule, incorporating existing data on the HLA alleles most frequently described in RA (HLA DR4).

The programs BcePred and ABCpred were used to select the B epitopes. The parameters and scores shown below were used as selection criteria.

- BcePred (http://www.imtech.res.in/raghava/bcepred/): The results are obtained using the default parameters, and a list of B epitopes sequences with an arginine at the end and another in the middle of the sequence is obtained.
- ABCpred (http://www.imtech.res.in/raghava/abcpred/ABC_submission.html): >0.75 was used as the threshold parameter, while the other parameters remained standard.

The ProPred programs were used to select the T epitopes; MHCPred version 2.0. Each of the parameters and scores shown below was used as selection criteria.

- NETMHCII (http://www.cbs.dtu.dk/services/NetMHCII/): The alleles described above were selected, and the program was run with standard parameters, considering that the threshold was −99.
- ProPred (http://www.imtech.res.in/raghava/propred/): A threshold of >7.5 was selected, along with the corresponding alleles, and the program was executed.

After the possible peptides were generated, they were organised by each of the programs used, and those that were identified by at least two of them and recognised as B and T epitopes were chosen. Subsequently, BLAST was performed for these peptides using the UniProt database tool (http://www.uniprot.org/blast/) to confirm specificity for *P. gulae*, *P. gingivalis* and human PAD. Once the peptides were chosen (1. native sequence, 2. with modifications (citrullination), and 3. random sequence [Figure S4]), their synthesis by the company GenScript® (GenScript USA Inc., 860 Centennial Ave., Piscataway, NJ 08854) was requested.

A solid-phase immunoassay (ELISA) was conducted for the detection of anti-PAD antibodies. For this, the peptides synthesised from *P. gulae* PAD were fixed in ELISA plates (Ref. 655061, Greiner Bio-one) at a concentration of 10 µg/well in carbonate–bicarbonate buffer (pH 11.0) at 4°C for 14–16 h. Subsequently, the sensitisation solution was discarded and 100 µL/well of PBS 1X–1% bovine serum albumin (BSA) solution (Ref.820451, Probumin (diagnostic grade) was placed in order to block non-specific sites. 1/100 and 1/200 dilutions of patient and control sera were made in PBS 1X–Tween 0.05%–BSA 1%, and 100 µL/well of the diluted serum were seeded on the plate and incubated for 1 h at 37ºC. Next, three 2-min washes were performed with PBS 1X–Tween 0.05% and then the HRP-labelled anti-IgG antibody was placed in a 1/10,000 dilution in PBS 1X–Tween 0.05%–BSA 1% and incubated for 1 h at room temperature. Next, washings were carried out under the same conditions as before, and 100 µL/well of the revealing solution in 1:1 dilution of TMB-KPL was added and allowed to react for 2 min at room temperature. Finally, the reaction was stopped using 100uL/well of stop solution (1M H2SO4), and the reading was performed on the TECAN infinite M200 pro device (Ref. 30050303, Austria) at a wavelength of 450 nm and with a correction of 620 m. The presence of anti-citrullinated peptide antibodies from *P. gulae* PAD was determined by the absorbance data of the wells sensitised with the peptides after subtracting the absorbance of the negative control wells without peptides.


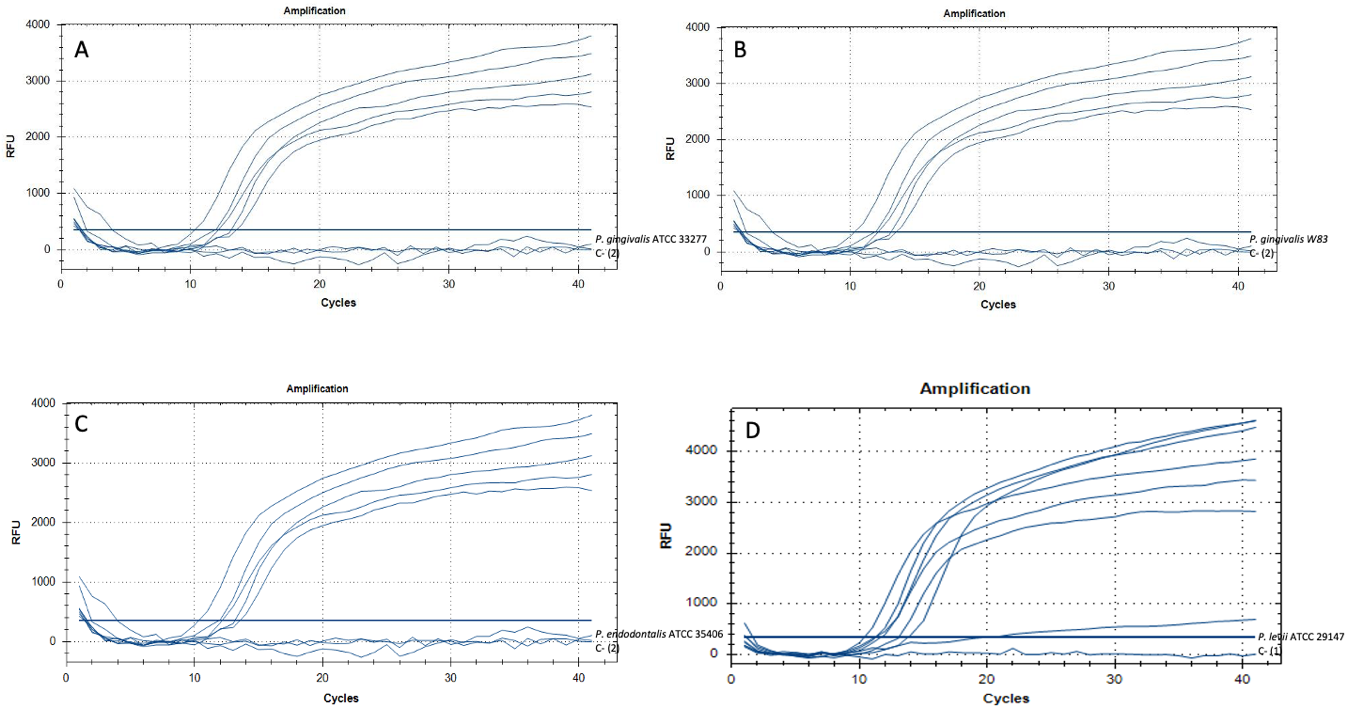


**Figure S2**. Amplification curves of four P. gulae primers and probe specificity experiments. qPCR. **a**. DNA from P. gulae ATCC 51700 and P. gingivalis ATCC 33277. **b**. DNA from P. gulae ATCC 51700 and P. gingivalis W83. **c**. DNA from P. gulae ATCC 51700 and P. endodontalis ATCC 35406. **d**. DNA from P. gulae ATCC 51700 and P. levii ATCC 29147. C-: negative control.


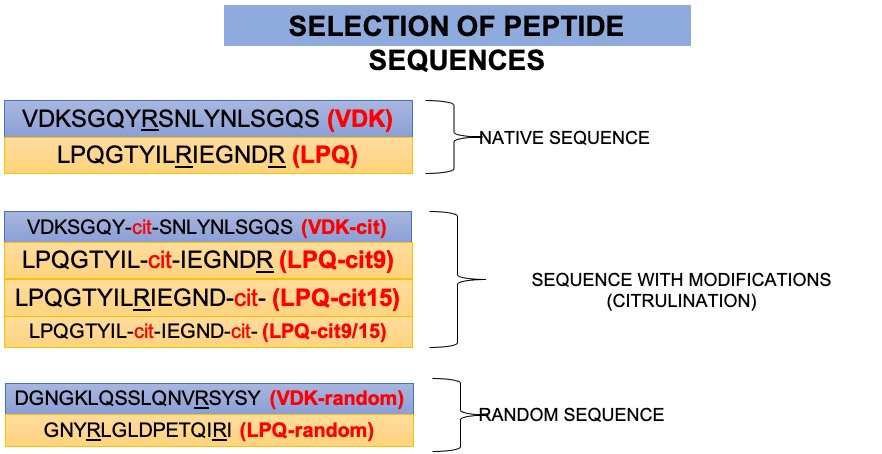


**Figure S3**. Selected sequences of PAD peptides from *P. gulae*.


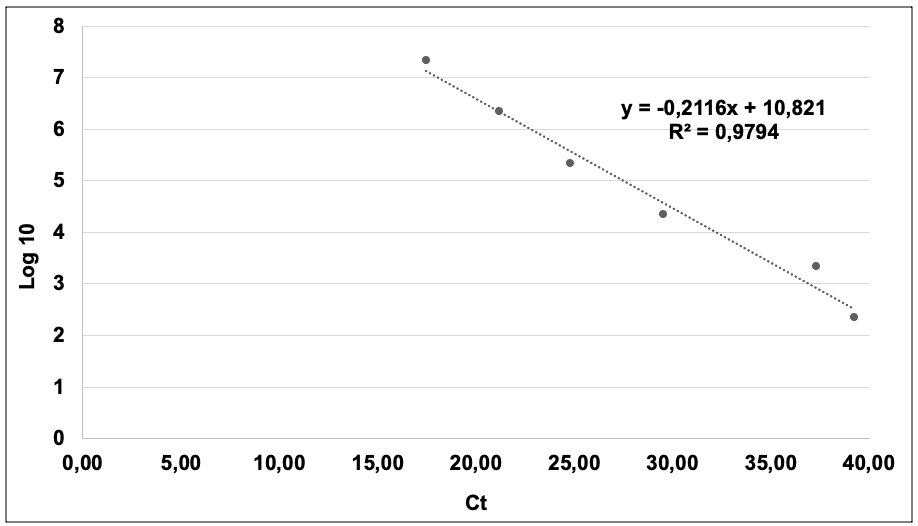


**Figure S4.** Quantification curves obtained with dilutions of DNA from *P. gulae* ATCC 51700 extracted from an inoculum of the bacterium with a known number of colony-forming units.

**Reference**

1. Senhorinho GN, Nakano V, Liu C, Song Y, Finegold SM, Avila-Campos MJ. Detection of Porphyromonas gulae from subgingival biofilms of dogs with and without periodontitis. Anaerobe. 2011;17(5), 257-258. doi: 10.1016/j.anaerobe.2011.06.002.
